# Dietary eggshell membrane modulates gut microbiota and alleviates AOM/DSS-induced colonic inflammation in mice

**DOI:** 10.1042/BSR20253696

**Published:** 2026-01-09

**Authors:** Wenqiang Yin, Sihui Ma, Maki Igarashi, Xuguang Li, Noriko Suzuki-Kemuyama, Yukio Hasebe, Hisanori Kato, Huijuan Jia

**Affiliations:** 1 Graduate School of Medicine and Faculty of Medicine, The University of Tokyo, Tokyo, Japan; 2 Graduate School of Agricultural and Life Sciences, The University of Tokyo, Tokyo, Japan; 3 Faculty of Sport Sciences, Waseda University, Tokorozawa, Japan; 4 Institute of Nutrition Sciences, Kagawa Nutrition University, Sakado, Japan; 5 Department of Nutritional Science and Food Safety, Faculty of Applied Bioscience, Tokyo University of Agriculture, Tokyo, Japan; 6 ALMADO Inc., Tokyo, Japan; 7 Department of Applied Nutrition, School of Nutrition, Kagawa Nutrition University, Sakado, Japan

**Keywords:** colitis-associated colorectal cancer, dietary intervention, eggshell membrane, gut microbiota, inflammation

## Abstract

Persistent intestinal inflammation and gut dysbiosis are key drivers of the progression from inflammatory bowel disease (IBD) to colitis-associated colorectal cancer (CAC). Eggshell membrane (ESM) has shown beneficial effects in alleviating IBD symptoms; however, its impact on inflammation-driven CAC remains unclear. This study investigated the effects of dietary ESM supplementation in an azoxymethane/dextran sulfate sodium (AOM/DSS)-induced CAC mouse model. Mice were fed either a control or ESM-supplemented diet for one month prior to CAC induction and continuing throughout the experiment. ESM supplementation significantly improved survival rates in AOM/DSS-induced CAC mice and reduced colitis severity, indicated by the down-regulation of the pro-inflammatory cytokines and M1-like macrophage markers in the colonic mucosa. In addition, ESM conferred systemic protective effects by alleviating liver inflammation and tissue damage, as evidenced by preserved hepatic structure and reduced immune cell infiltration. Importantly, ESM significantly restored cecal microbiota composition disrupted by AOM/DSS, reducing pathogenic bacteria such as *Escherichia–Shigella*, while enriching beneficial bacteria such as Firmicutes and Muribaculaceae. These compositional shifts were accompanied by predicted correction of dysregulated microbial metabolic pathways, reflecting a restoration of microbial functional balance. Overall, these findings demonstrate that ESM alleviates multi-organ inflammation and restores microbial balance in CAC, highlighting its potential as a dietary strategy to mitigate chronic inflammation and its systemic consequences.

## Introduction

Inflammatory bowel disease (IBD), including Crohn’s disease and ulcerative colitis, is characterized by chronic immune-mediated intestinal inflammation that severely affects patient quality of life [1,2]. Persistent inflammation markedly increases the risk of developing colitis-associated colorectal cancer (CAC), a subtype of colorectal cancer linked specifically to chronic intestinal inflammation [3,4]. The gut microbiota plays a pivotal role in IBD pathogenesis by regulating immune responses and maintaining epithelial integrity [5,6]. Under normal conditions, commensal gut bacteria contribute to digestion, barrier maintenance, and immune regulation [7]. Germ-free mice typically do not develop colitis, whereas colonization with commensal bacteria can trigger inflammatory changes [8]. However, IBD patients often exhibit gut dysbiosis, characterized by an imbalance in microbial composition and diversity, leading to mucosal inflammation and disease progression [9,10]. This dysbiosis, influenced by environmental factors such as diet and stress, can further exacerbate intestinal barrier dysfunction, allowing bacterial translocation to other organs, including the liver [11]. Therefore, restoring microbial balance and enhancing intestinal barrier integrity are crucial for mitigating both local and systemic complications of IBD, including CAC development [12,13]. Dietary interventions represent a promising strategy for modulating gut microbiota and mitigating IBD-associated complications. A high-calorie diet rich in fat and carbohydrates promotes pro-inflammatory bacterial genera, including *Bacteroides* and *Prevotella*, whereas high dietary fiber intake correlates positively with increased microbial diversity and improved gut health [14,15]. Eggshell membrane (ESM), a natural source of bioactive compounds, has previously demonstrated anti-inflammatory effects and beneficial modulation of gut microbiota in interleukin-10 knockout mice and in dextran sulfate sodium (DSS)–induced colitis models [16–18]. In prior studies, dietary ESM improved intestinal barrier function, reduced inflammatory responses, increased microbial diversity, and decreased pathogenic bacterial populations, such as Enterobacteriaceae and *Escherichia coli*. Additionally, ESM increased short-chain fatty acids (SCFAs) production, improved survival rates, and restored microbial homeostasis, particularly by normalizing the Firmicutes/Bacteroidetes ratio and enhancing beneficial taxa such as *Ruminococcus* and Bacteroidales [16–18]. Collectively, these findings suggest that ESM effectively modulates gut microbiota composition and immune responses, thus promoting intestinal health.

Given these demonstrated benefits, we hypothesized that dietary ESM supplementation could affect the progression of CAC by modulating intestinal inflammation and microbial composition. The current study aimed to clarify the effects of ESM on CAC progression, specifically examining its influence on gut microbiota and inflammation in a well-established mouse model of azoxymethane/dextran sulfate sodium (AOM/DSS)-induced CAC.

## Methods and materials

### Animals and experimental design

All animal experiments were approved by and carried out in strict accordance with the guidelines of the Animal Usage Committee of the Graduate School of Agricultural and Life Sciences at the University of Tokyo (Approval No. P21-039). Six-week-old male C57BL/6J mice were purchased from CLEA Japan (Tokyo, Japan) and were housed in vinyl isolators under a temperature of 22 ± 1°C and a humidity of 60 ± 5% in a 12 h/12 h light/dark cycle (08:00 to 20:00). After a week of acclimation to the prescribed environmental conditions, mice were randomly assigned to either a control normal AIN-93G diet group (CAC-ND) or an ND supplemented with 8% ESM diet group (CAC-ESM) (Supplementary Table S1). After one month, colitis-associated CAC was induced in both groups using the AOM/DSS protocol. Throughout the CAC induction period, the mice continued to receive their respective CAC-ND or CAC-ESM diets. All the animals were anesthetized with 4% v/v inhaled isoflurane at the end of the experiment, and cervical dislocation was performed.

### Induction of colitis-associated CAC

CAC was induced using a modified AOM/DSS protocol as described previously [19]. Briefly, mice in the CAC groups received an intraperitoneal injection of AOM (10 mg/kg body weight; Sigma-Aldrich, MO, U.S.A.) at the beginning of the experiment. Seven days after AOM administration, mice were provided with drinking water containing 2.5% DSS (molecular weight 36–50 kDa; MP Biomedicals, OH, U.S.A.) *ad libitum* for seven days, followed by 14 days of regular drinking water. This DSS cycle was repeated four times. Mice in the control group received an equivalent volume of saline injection instead of AOM and were maintained on regular drinking water throughout the study.

### Disease activity index assessment

The disease activity index (DAI) was assessed in AOM/DSS-induced colitis based on established parameters indicative of inflammation, including weight loss, stool consistency, and rectal bleeding, as previously described [20]. Each parameter was assessed daily starting from the first day of DSS treatment, and the DAI was calculated as the average of these three scores [16]. Body weight loss was scored as follows: 0, <5% weight loss; 1, 5–9% weight loss; 2, 10–14% weight loss; 3, 15–19% weight loss; and 4, ≥20% weight loss. Stool consistency was evaluated using the following scale: 0, normal; 1, soft but still formed; 2, soft, formed stools adhering to the anus; 3, semi-liquid or occasional diarrhea; and 4, severe diarrhea. Rectal bleeding was assessed as follows: 0, negative for occult blood (Hemoccult) and normal stool; 1, negative for occult blood but soft stool; 2, positive for occult blood; 3, visible blood traces in the stool; and 4, gross rectal bleeding. All scoring was performed in a blinded manner to minimize observer bias.

### Tissue collection and processing

Tissues were collected from all mice, either at the end of the experiment (week 10, after the fourth DSS cycle) or at the time of death before the endpoint, using the same standardized procedures as for surviving mice. The fourth DSS cycle was selected as the final time point because chronic inflammation and neoplastic transformation are well established at this stage. All collected samples were included in subsequent analyses to ensure representation of the entire study population. The length of the colon was measured as the distance from the cecum to the proximal rectum. Liver and colon tissues were collected and processed for histological analysis, RNA extraction, and 16S rRNA gene sequencing.

### Histological analysis

The liver and colon were harvested as described previously [16]. The tissues were embedded in optimal cutting temperature (OCT) compound (Sakura Finetek, CA, U.S.A.) and snap-frozen in liquid nitrogen. The embedded tissue samples were then sectioned at 5 μm thickness and stained with hematoxylin and eosin (H&E). Images were acquired using an Olympus BX51 microscope (Olympus Optical, Tokyo, Japan). Inflammation and tissue injury were evaluated semi-quantitatively based on morphological changes. For H&E sections of colons, scores were assigned according to the extent of inflammatory cell infiltration in the mucosa and submucosa and the degree of mucosal structural disruption. For H&E sections of livers, scores were assigned according to the inflammatory cell infiltration, centrilobular necrosis, centrilobular microvascular change, and sinusoidal dilation. Each parameter was graded on a four-point scale: 0 (negative), 1 (slight), 2 (moderate), and 3 (severe). For each mouse, more than five non-overlapping fields were evaluated, and the mean score per parameter was used for analysis. Group data are presented as frequency distributions to illustrate the range of severity within each group. The number and size of tumors in the colon were measured, and the degree of dysplasia was assessed by a pathologist blinded to the treatment groups.

### RNA extraction and quantitative reverse transcription PCR (qRT-PCR)

Total RNA was extracted from liver and colonic mucosa tissue samples using the E.Z.N.A.® Total RNA Kit II (Omega Bio-Tek, Norcross, GA, U.S.A.) following the manufacturer’s instructions. RNA concentration and purity were assessed by NanoDrop One, ensuring A260/A280 ratios between 1.8 and 2.0. Reverse transcription to synthesize cDNA was performed using the PrimeScript^TM^ RT Master Mix (Perfect Real Time; Takara Bio Inc., Otsu, Japan). qRT-PCR was performed using the Thermal Cycler Dice Real Time System TP800 (Takara Bio Inc., Otsu, Japan) with specific primers for target genes (Supplementary Table S2). The conditions for the qPCR cycling were as follows: 95°C for 30 s, followed by 40 cycles of 95°C for 5 s, and 60°C for 30 s. Additionally, dissociation was examined at 95°C for 15 s, 60°C for 30 s, and 95°C for 15 s. Each sample was run in triplicate. mRNA expression levels were normalized to *Actb* (β actin) for liver tissue and ribosomal protein lateral stalk subunit P1 (*Rplp1*) for colonic mucosa. Gene expression was quantified using the 2^-ΔΔCt^ method, and results were expressed as fold-change values relative to the control group.

### 16S rRNA gene sequencing

DNA extraction from cecal contents was performed using the QIAamp Stool Mini Kit (Qiagen, Hilden, Germany) as described previously [16]. The variable regions 3 and 4 of the 16S rRNA were amplified with the primers 5′-CCTACGGGNGGCWGCAG-3′ and 5′-GACTACHVGGGTATCTAATCC-3′, which were modified to include Illumina adapters and barcode sequences for subsequent sequencing. Library size and quantification analysis were performed using the Agilent 2100 Bioanalyzer (Agilent Technologies, Santa Clara, CA, U.S.A.). All libraries were pooled in a single Illumina MiSeq run (MiSeq Reagent Kit V3, 600 cycles, Illumina, San Diego, CA, U.S.A.).

### Analysis of the bacterial microbiome

Sequence data were analyzed using QIIME2 (version 2024.5) [21]. The DADA2 plugin was employed for quality control and denoising of sequencing reads. During denoising, forward reads were truncated at 300 bases and reverse reads at 240 bases to remove low-quality regions. Taxonomy classification was visualized using QIIME2 View, with taxonomic bar plots generated at the phylum, family, and genus levels. Alpha diversity was assessed by calculating the Shannon diversity index using the phyloseq package (v1.46.0) in R (version 4.3.3). Beta diversity was evaluated using Bray–Curtis distance metrics computed with the vegan package (v2.6-8), followed by principal coordinate analysis (PCoA) and visualization with ggplot2 in R. Functional pathway prediction was performed using PICRUSt2 [22] following instructions.

### Statistical analysis

All data are expressed as mean ± standard error of the mean (SEM). Statistical analyses were performed using GraphPad Prism (version 10.0.0, GraphPad Software). The specific statistical tests applied are indicated in the corresponding figure legends. Differences were considered statistically significant at *P*<0.05.

## Results

### ESM attenuates disease severity in a CAC mouse model

**Figure 1:**
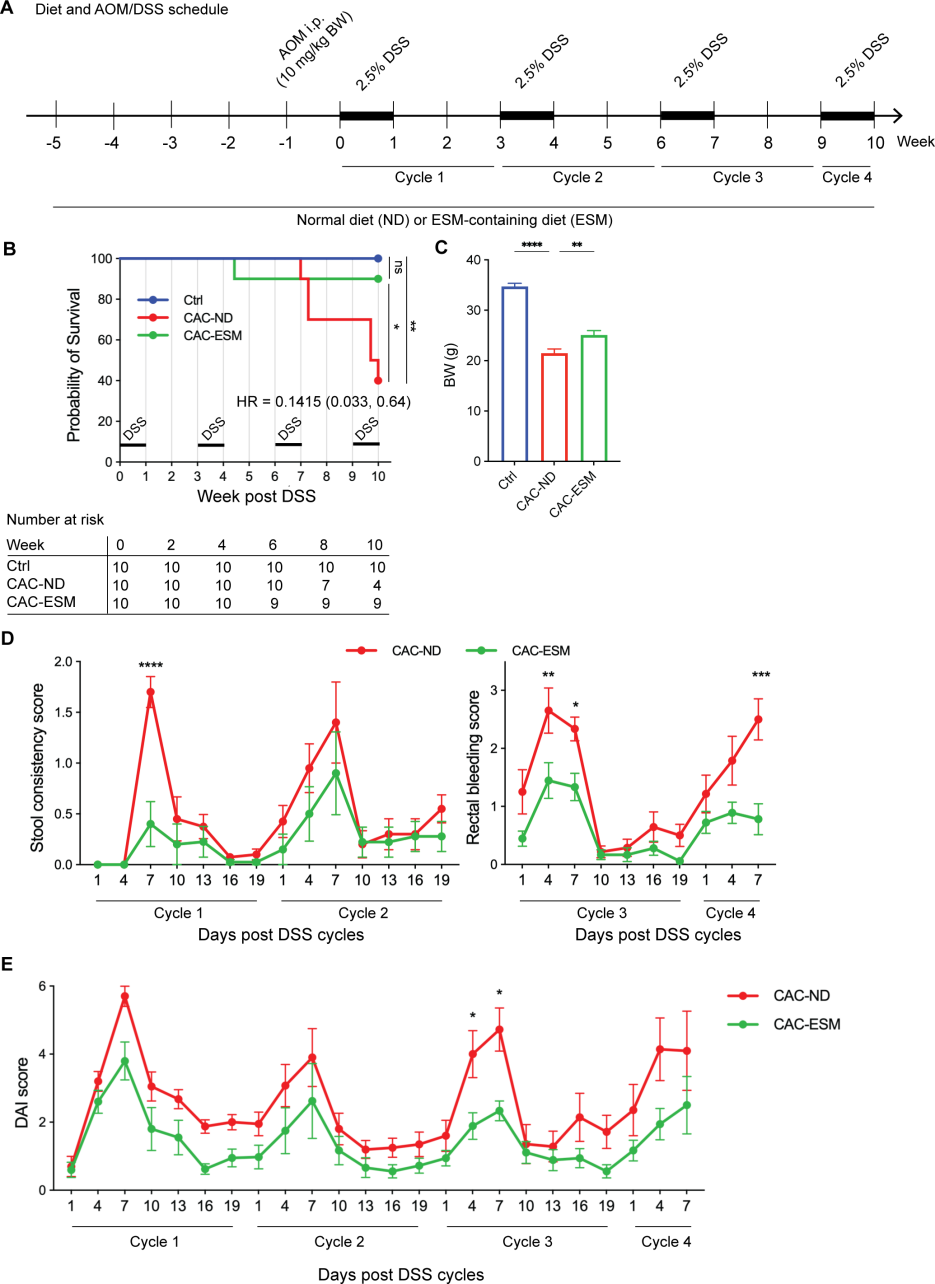
ESM supplementation improves survival and alleviates disease severity in AOM/DSS-induced CAC mice. (**A**) Schematic representation of the AOM/DSS-induced colitis-associated CAC model and the experimental timeline. Mice were injected with AOM followed by four cycles of 2.5% DSS administration in drinking water to induce CAC. The experimental groups included mice fed a normal diet (CAC-ND) and those receiving an ESM-supplemented diet (CAC-ESM). (**B**) Kaplan–Meier survival analysis showing the percentage of surviving mice in the Ctrl, CAC-ND, and CAC-ESM groups over 10 weeks. The hazard ratio (HR) indicates a significantly lower risk of death in the CAC-ESM group compared with the CAC-ND group (HR=0.1451, 95% confidence interval: 0.033–0.64). *P*-value was calculated using the log-rank (Mantel-Cox) test. At week 0, ten mice were assigned to each group. The number at risk (surviving mice) at weeks 2, 4, 6, 8, and 10 is indicated below the plot. (**C**) Body weight (BW) was recorded at the endpoint for each mouse. Statistical analysis was performed using one-way ANOVA followed by Tukey’s test. (**D**) Stool consistency and rectal bleeding scores were measured during the indicated period. (**E**) DAI was assessed throughout the experiment. Statistical analysis was performed using two-way ANOVA with Sidak’s multiple comparison correction (**D–E**). Data are expressed as mean ± SEM. *P<0.05, **P<0.01, ***P<0.001, and ****P<0.0001. ns, not significant. Symbols represent individual mice. *n*=4–10 per group. DAI, disease activity index.

To assess the potential protective effects of dietary ESM supplementation against CAC, we used a well-established AOM/DSS-induced CAC mouse model, in which mice were fed either a normal diet (CAC-ND) or an ESM-containing diet (CAC-ESM) (Figure 1A). By week 10, more than 50% of CAC-ND mice had died, reflecting the lethality of AOM/DSS treatment (Figure 1B). In contrast, ESM substantially improved survival, with over 90% of CAC-ESM mice remaining alive (Figure 1B). These results demonstrate a protective effect of ESM supplementation against CAC-related mortality. Furthermore, body weight loss, a key indicator of disease severity, was significantly attenuated in CAC-ESM mice compared with CAC-ND mice (Figure 1C). ESM supplementation also improved stool consistency and reduced rectal bleeding, as reflected by lower DAI scores (Figure 1D–E). Collectively, these results suggest that dietary ESM supplementation effectively reduces CAC severity.

### ESM attenuates colonic inflammation and modulates macrophage polarization independently of tumor suppression

**Figure 2:**
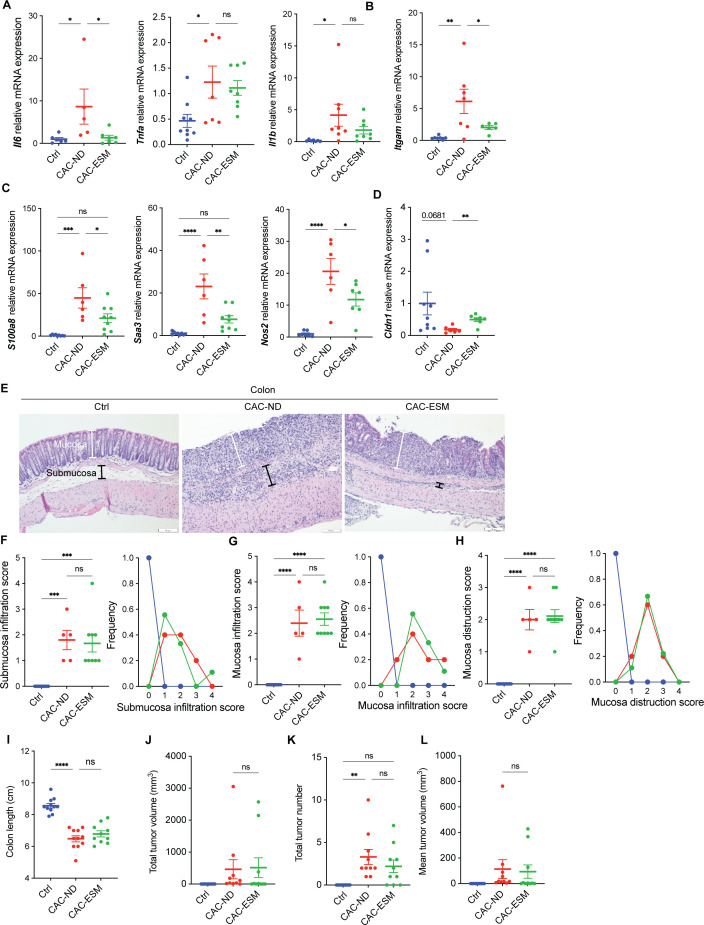
The effects of ESM on colitis-associated colonic inflammation and tumor burden in AOM/DSS-induced CAC mice. (**A–D**) Relative mRNA expression in colonic mucosa. Pro-inflammatory cytokines *Il1b*, *Tnfa,* and *Il6* (**A**); the granulocyte and macrophage marker, *Itgam* (CD11b) (**B**); M1-like macrophage markers, *S100a8*, *Saa3,* and *Nos2* (**C**); the epithelial integrity gene, *Cldn1* (**D**). (**E**) Representative H&E-stained images of colon tissue. White and black lines indicate the boundaries of the mucosa and submucosa, respectively. Scale bar=100 μm. (**F–H**) Histological scoring of H&E-stained colon sections for submucosa infiltration (**F**), mucosa infiltration (**G**), and mucosal destruction (**H**), with the frequency of each score in the indicated groups. (**I**) Colon length on the final day of the fourth DSS cycle. (**J–L**) Tumor burden analysis showing total tumor volume (**J**), total tumor number (**K**), and mean tumor volume (**L**). All data are from control (Ctrl), CAC-ND, and CAC-ESM mice. Analyses were performed for all mice, either at the time of death or at the 10-week endpoint for surviving mice. Statistical analysis was performed using one-way ANOVA followed by Tukey’s test. Data are expressed as mean ± SEM. *P<0.05, **P<0.01, ***P<0.001, and ****P<0.0001. Symbols represent individual mice. *n*=6–10 per group. ns, not significant.

To investigate the mechanisms underlying the protective effects of ESM in CAC, we assessed colonic inflammation. ESM supplementation reduced the expression of the pro-inflammatory cytokines *Il6*, but not *Tnfa* and *Il1b* in the colonic mucosa (Figure 2A). Additionally, the reduced *Itgam* (CD11b) expression was observed in the CAC-ESM group, reflecting decreased infiltration of granulocytes and macrophages (Figure 2B). ESM also down-regulated the expression of genes associated with M1-like macrophages (*S100a8*, S100 calcium-binding protein A8; *Saa3*, serum amyloid A3; and *Nos2*, nitric oxide synthase 2) (Figure 2C), whereas expression of genes associated with M2-like macrophages (*Arg1*, arginase 1; *Cd163* and *Mrc1*, mannose receptor C-type 1) remained unchanged (Supplementary Figure S1A). These results suggest that ESM exerts anti-inflammatory effects in the colon by suppressing the M1-like macrophage polarization and expression of the pro-inflammatory cytokine. To evaluate the impact of ESM on intestinal barrier function, we investigated the expression of key tight junction genes in the colonic mucosa. The expression of *Cldn1* (claudin-1) was significantly higher in the CAC-ESM mice than in the CAC-ND mice (Figure 2D), whereas no significant differences were observed in *Ocln* (occludin) or *Tjp1* (ZO1) expression among groups (Supplementary Figure S1B). Despite these effects, ESM did not significantly affect features related to tumor progression, including histological infiltration scores (Figure 2E–H) and colon shortening (Figure 2I), nor did it show a significant tumor-suppressive effect (Figure 2J–L). These observations suggest that the beneficial effects of ESM in this CAC model are primarily mediated by its anti-inflammatory actions rather than direct anti-tumor effects.

### ESM protects against liver damage and inflammation by preserving tissue architecture

**Figure 3:**
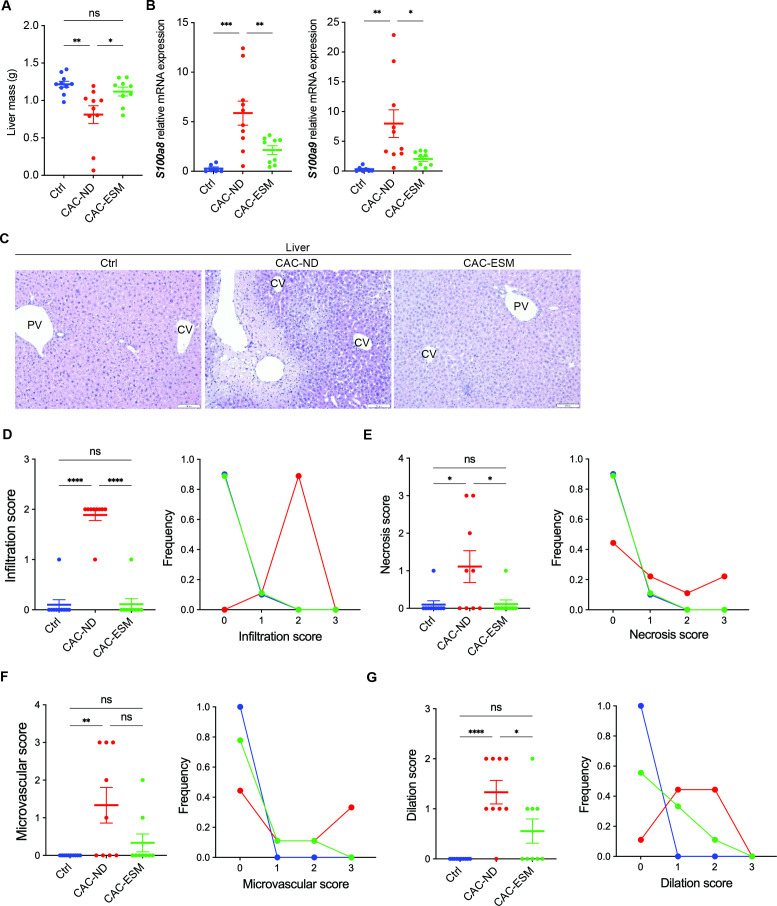
ESM protects against liver damage and inflammation in the AOM/DSS model of CAC. **(A**) Liver mass measured on the final day of the fourth AOM/DSS cycle. (**B**) Relative hepatic *S100a8* and *S100a9* mRNA expression. (**C**) Representative H&E-stained images of liver tissue. Scale bar=100 μm. (**D–G**) Histological scoring of H&E-stained liver sections for immune cell infiltration (**D**), necrosis (**E**), microvascular changes (**F**), and vascular dilation (**G**), with the frequency of each score in the indicated groups. All data are from control (Ctrl), CAC-ND, and CAC-ESM mice. Statistical analysis was performed using one-way ANOVA followed by Tukey’s test. Data are expressed as mean ± SEM. *P<0.05, **P<0.01, ***P<0.001, and ****P<0.0001. Symbols represent individual mice. *n*=7–10 per group. ns, not significant; PV, portal vein; CV, central vein.

Chronic intestinal inflammation can disrupt gut–liver interactions, resulting in hepatic injury through systemic inflammation and microbial translocation [23]. Therefore, in addition to assessing colonic pathology, examining liver involvement provides critical insight into the systemic manifestations of CAC. At week 10, AOM/DSS-induced CAC led to a significant reduction in liver mass in CAC-ND mice, indicating liver injury, which was partly reversed by ESM supplementation (Figure 3A). The increased expression of *S100a8* and *S100a9* observed in CAC-ND mice was suppressed in CAC-ESM mice (Figure 3B), suggesting that ESM alleviates myeloid-driven inflammation within the liver. In contrast, the hepatic expression levels of pro-inflammatory cytokines *Tnfa*, *Il1b,* and *Il6* were not significantly changed among groups (Supplementary Figure S1C). These findings indicate that colonic damage induced by AOM/DSS induces a sustained, low-grade hepatic inflammatory state rather than acute liver injury. Histological analysis confirmed severe hepatic damage in the CAC-ND group, characterized by disrupted hepatic architecture, central vein dilation, and hepatocyte degeneration (Figure 3C). These pathological changes were notably improved in the CAC-ESM group (Figure 3C). ESM supplementation significantly decreased immune cell infiltration, necrotic damage, and microvascular alterations in the liver (Figure 3D–G). Thus, ESM supplementation effectively mitigates hepatic injury associated with CAC.

### ESM restores cecal microbiota composition altered by AOM/DSS

**Figure 4:**
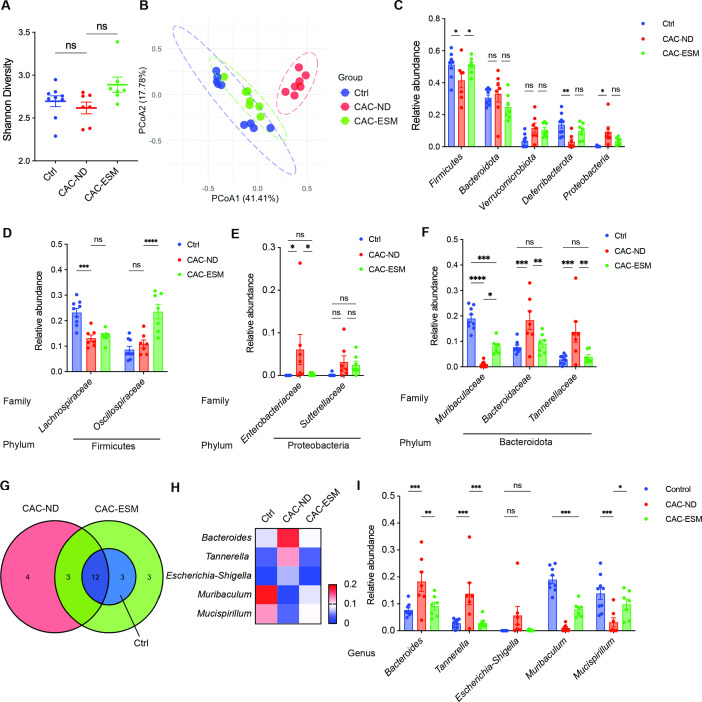
ESM partially restores cecal microbiota composition altered by AOM/DSS treatment. **(A and B**) Alpha diversity (Shannon index) (**A**) and beta diversity (principal coordinate analysis; PCoA) (**B**) of cecal microbiota at week 10. (**C**) Relative abundance of the top five most abundant bacterial phyla in cecal contents. (**D–F**) Relative abundance of the bacterial families of Firmicutes (**D**), Proteobacteria (**E**) and Bacteroidota (**F**) in cecal contents. (**G**) Venn diagram illustrating bacterial taxa with >1% relative abundance shared between groups. (**H and I**) Heatmap (**H**) and statistical analysis (**I**) of the relative abundance of indicated genera. All data are from control (Ctrl), CAC-ND, and CAC-ESM mice. Statistical analysis was performed using two-way ANOVA followed by Tukey’s test. Data are expressed as mean ± SEM. *P<0.05, **P<0.01, ***P<0.001, and ****P<0.0001. Symbols represent individual mice. *n*=7–9 per group. ns, not significant; PCoA, principal coordinate analysis.

To investigate whether the protective effects of ESM involved changes in microbiota, we analyzed microbial composition using 16S rRNA sequencing of cecal contents, since the murine cecum is a major fermentation site and shows the most pronounced microbial responses to environmental factors [24–26]. The Shannon diversity (alpha diversity) analysis revealed no significant differences between groups, suggesting that overall microbial richness and evenness were not affected by ESM supplementation (Figure 4A). However, beta diversity analysis clearly differentiated the microbiota composition between CAC-ND and control mice, indicating a substantial shift in microbiota composition following AOM/DSS treatment (Figure 4B). Notably, ESM supplementation partially restored microbiota balance, as microbiota profiles in the CAC-ESM group clustered more closely with those of the control mice (Figure 4B).

At the phylum level, the five major phyla, Firmicutes, Bacteroidota, Verrucomicrobiota, Deferribacterota, and Proteobacteria, together accounted for more than 90% of the total taxa (Supplementary Figure S2A). Among these phyla, the relative abundances of Firmicutes and Deferribacterota were significantly decreased, whereas Proteobacteria was increased in CAC-ND mice compared with the control group (Figure 4C). These alterations were reversed by ESM supplementation, although some did not reach statistical significance (Figure 4C).

At the family level, the top ten families accounted for nearly 90% of the total taxa, with the top three families (Lachnospiraceae, Bacteroidaceae, and Oscillospiraceae) together comprising approximately 50% of the total taxa (Supplementary Figure S2B). Among them, the families Lachnospiraceae and Oscillospiraceae (members of the phylum Firmicutes) are known to produce SCFAs, supporting epithelial integrity and suppressing inflammation [27]. Although Lachnospiraceae showed no significant recovery in CAC-ESM mice, the marked increase of Oscillospiraceae compared with both control and CAC-ND groups (Figure 4D) may compensate for the loss of Lachnospiraceae, suggesting that ESM helps restore SCFA production in the gut. The Proteobacteria phylum comprises many facultative anaerobic and potentially pathogenic taxa, including the families Enterobacteriaceae and Sutterellaceae. ESM supplementation markedly reduced the AOM/DSS-induced increase in Enterobacteriaceae (Figure 4E), indicating a recovery from inflammation-associated dysbiosis [28]. Although the relative abundance of the phylum Bacteroidota did not differ among groups (Figure 4C), ESM supplementation significantly rebalanced the relative abundances of its families (Figure 4F), including Muribaculaceae, Bacteroidaceae, and Tannerellaceae, which have been associated with anti-inflammatory activity in the gut. However, their roles are strain- and context-dependent [29,30].

At the genus level, we focused on taxa with a relative abundance greater than 1% in each group (Supplementary Figure S3A–C). The CAC-ESM group shared all genera with the control group (Figure 4G). Particularly, the abundances of *Bacteroides* (phylum Bacteroidota, family Bacteroidaceae), *Tannerella* (phylum Bacteroidota, family Tannerellaceae), and *Escherichia–Shigella* (phylum Proteobacteria, family Enterobacteriaceae) were markedly elevated in CAC-ND mice, reflecting an inflammation-associated dysbiosis, but were decreased to near-control levels by ESM supplementation (Figure 4H–I). Conversely, the beneficial genera *Mucispirillum* (phylum Deferribacterota, family Deferribacteraceae) and *Muribaculum* (phylum Bacteroidota, family Muribaculaceae) [31,32], which were depleted in CAC-ND mice, rebounded toward normal levels following ESM treatment (Figure 4H–I), suggesting a reestablishment of microbial homeostasis.

Changes in cecal microbiota composition can trigger functional shifts that promote the development of IBD. To further clarify the functional impact of microbiota alterations, we conducted pathway prediction analysis based on 16S rRNA sequencing data. Pathway analysis demonstrated significant enrichment of microbial metabolic pathways involved in aromatic biogenic amine degradation and tRNA processing in the CAC-ND group (Supplementary Figure S3D), suggesting dysregulated microbial metabolism. Collectively, these findings suggest that ESM substantially restores microbial balance toward a normal state and modulates the predicted functional capacity of the cecal microbiota, which may contribute to its protective effects in the pathogenesis of CAC.

## Discussion

In this study, we investigated the protective effects of dietary ESM supplementation in a mouse model of colitis-associated CAC induced by AOM/DSS. This model captures key pathological features of CAC development, including chronic intestinal inflammation and cecal microbiota dysbiosis. Our findings demonstrate that ESM supplementation significantly improved survival rates, suggesting its potential to attenuate disease progression under chronic inflammatory conditions. In the colon, ESM attenuated inflammation, marked by reduced expression of some of the pro-inflammatory cytokines and M1 macrophage markers. Moreover, ESM exerted protective effects against hepatic injury, evidenced by preserved hepatic architecture, reduced inflammatory cell infiltration, and diminished hepatic necrosis. Furthermore, ESM partially restored cecal microbiota composition disrupted by AOM/DSS, alongside correction of microbial functional imbalances. Together, these findings indicate that ESM mitigates inflammation and gut dysbiosis in CAC mice, supporting its potential as a new dietary intervention to improve the systemic consequences.

Consistent with previous reports, the expression of several pro-inflammatory cytokines, such as *Il1b*, *Il6,* and *Tnfa*, was elevated following AOM/DSS treatment. However, not all these cytokines were significantly suppressed by ESM supplementation. Instead, several M1-like or inflammation-associated genes (*S100a8*, *Saa3*, *Nos2*) were down-regulated in the CAC-ESM group. These findings suggest that ESM may not directly inhibit the expression of these cytokines but rather attenuate M1 macrophage polarization, thereby mitigating mucosal inflammation at a broader transcriptional level. Moreover, while ESM improved survival, it was insufficient to significantly reduce overall tumor burden in this model, indicating that ESM primarily modulates the inflammatory microenvironment, particularly during the M1-dominant phase, rather than exerting direct cytotoxic effects on tumor cells. Our analyses were mainly conducted at week 10, the end of the fourth DSS cycle. Because M1 macrophages are generally dominant during the early stage of inflammation, the M1-mediated effects on tumorigenesis that might be suppressed by ESM may not have been captured in this study. In addition, it is possible that the lack of a tumor-suppressive effect resulted from survival bias, as more ESM-treated mice reached the 10-week endpoint. To fully elucidate the potential impact of ESM on tumorigenesis, analyses at earlier stages (before the third DSS cycle) are needed. Such approaches might clarify the potential of ESM as an adjunct dietary intervention in CAC management.

Beyond the colon, ESM also protected against hepatic pathology in CAC mice. Specifically, ESM-treated mice exhibited improved hepatic histological architecture, reduced immune cell infiltration, and lower levels of necrotic injury. Given that chronic colonic inflammation disrupts gut–liver interactions, potentially facilitating bacterial translocation and systemic inflammation [33], our findings suggest that ESM may enhance intestinal barrier integrity and thus limit liver injury. Additionally, the effect of ESM on the hepatic tumor microenvironment remains to be clarified. Future studies should directly evaluate intestinal barrier integrity by assessing markers of intestinal permeability and quantifying bacteria in the liver, as well as investigate whether ESM alters susceptibility to tumor metastasis, providing further mechanistic insights into its protective roles.

Our study further demonstrated that ESM partially restored cecal microbiota composition disrupted by AOM/DSS. Consistent with human IBD, a substantial imbalance of major phyla, including Firmicutes and Proteobacteria [34], was also observed in CAC-ND mice. ESM supplementation restored the balance of these phyla, suggesting that ESM promotes the recovery of commensal bacteria disrupted by AOM/DSS treatment. Notably, *Escherichia–Shigella*, a member of Proteobacteria frequently enriched in colorectal cancer patients [35], increased to more than 1% in CAC-ND mice but was reduced to negligible levels in the ESM group. The phylum Firmicutes includes many well-known SCFA producers, and SCFAs such as butyrate, propionate, and acetate contribute to approximately 10% of total dietary energy in humans [34]. These metabolites play key roles in maintaining epithelial barrier integrity and modulating inflammation [36]. We found that Firmicutes was the dominant phylum in healthy control and CAC-ESM mice, albeit at lower abundance in CAC-ND mice, indicating their importance in sustaining gut homeostasis. In contrast, the second most abundant phylum, Bacteroidota, showed no significant difference among groups. Consequently, the Firmicutes-to-Bacteroidota (F/B) ratio was increased in ESM-treated mice (data not shown), which may reflect an enhanced capacity to ferment dietary polysaccharides into SCFAs [34]. Therefore, ESM likely contributes to the re-establishment of microbial equilibrium and SCFA production, thereby improving barrier function and promoting immune homeostasis. These beneficial effects may occur through microbiota-dependent mechanisms or through bioactive components in ESM, such as glycosaminoglycans and peptides, which can exert immunomodulatory functions independently of microbiota modulation. Functional pathway analysis revealed that AOM/DSS induced significant disruptions in microbial metabolic pathways, including pathways involved in aromatic biogenic amine degradation and tRNA processing. ESM supplementation attenuated these changes, restoring microbial functional profiles closer to those of healthy controls. Given the critical role of microbial metabolism in IBD pathogenesis, the observed restoration of microbial functional pathways may contribute to the anti-inflammatory effects of ESM. Nevertheless, because pathway analysis infers function from 16S rRNA-based taxonomy rather than direct biochemical measurements, these results should be interpreted as predictive. Future studies integrating metagenomic and targeted metabolomic analyses of key microbial metabolites, such as SCFAs, bile acids, and indole derivatives, will be essential to validate whether the observed microbial changes correspond to actual functional outcomes.

Collectively, this study provides evidence for the protective potential of ESM on the colon, liver, and gut microbiota, leading to significantly improved survival. A possible mechanism underlying these effects is proposed to involve modulation of the gut–liver inflammatory axis. In the AOM/DSS model, sustained intestinal inflammation impairs epithelial barrier integrity and induces microbiota dysbiosis, which in turn promotes systemic inflammation and liver injury. ESM helps restore microbial homeostasis, particularly by rebalancing SCFA-producing phyla, which directly or indirectly contribute to reduced inflammation and maintenance of intestinal barrier integrity, thereby attenuating hepatic inflammation and systemic immune activation that drive colorectal cancer progression. Nevertheless, a couple of general limitations should be noted. Although the AOM/DSS mouse model captures key features of human CAC, it does not fully reflect the clinical heterogeneity of the disease. In addition, because this study was conducted exclusively in male mice, and given the well-documented influence of sex differences on inflammation and microbiota composition, the generalizability of the findings should be interpreted with caution. Despite these limitations, ESM shows promise as an adjunct dietary strategy for managing CAC-related inflammation and dysbiosis.

## Supplementary material

online supplementary material 1.

## Data Availability

Data supporting the findings of this study are available upon reasonable request. The 16S rRNA sequence data generated have been deposited in a public repository under BioProject ID PRJNA1281723.

## Abbreviations

AOM/DSS: azoxymethane/dextran sulfate sodium
Arg1: arginase 1
CAC: colitis-associated colorectal cancer
Cd163: CD163 molecule
DAI: disease activity index
ESM: eggshell membrane
H&E: hematoxylin and eosin
IBD: inflammatory bowel disease
Mrc1: mannose receptor C-type 1
Nos2: nitric oxide synthase 2
SCFAs: short-chain fatty acids
S100a8: S100 calcium-binding protein A8
Saa3: serum amyloid A3
qRT-PCR: quantitative reverse transcription PCR
