# Supplementary material for: Dietary eggshell membrane modulates gut microbiota and alleviates AOM/DSS-induced colonic inflammation in mice
Source: Biochem J. 2026 Jan 9;46(Pt 1):BSR20253696. doi: 10.1042/BSR20253696 (PMC12905501; doi:10.1042/BSR20253696)
Supplement: online supplementary material 1. [file bcj-46-1-BSR20253696-s001.docx]

**Supplementary material**

**Figure S1**: Effects of ESM supplementation on the expression of colonic mucosal M2-like macrophage markers, epithelial tight junction genes, and hepatic inflammatory cytokines.

**Figure S2**: ESM supplementation modulates cecal microbiota composition at the phylum and family levels.

**Figure S3**: ESM supplementation rescues microbiome disruption in CAC mice.

**Table 1**: Diet composition.

**Table 2**: Primers for real-time PCR analysis

**
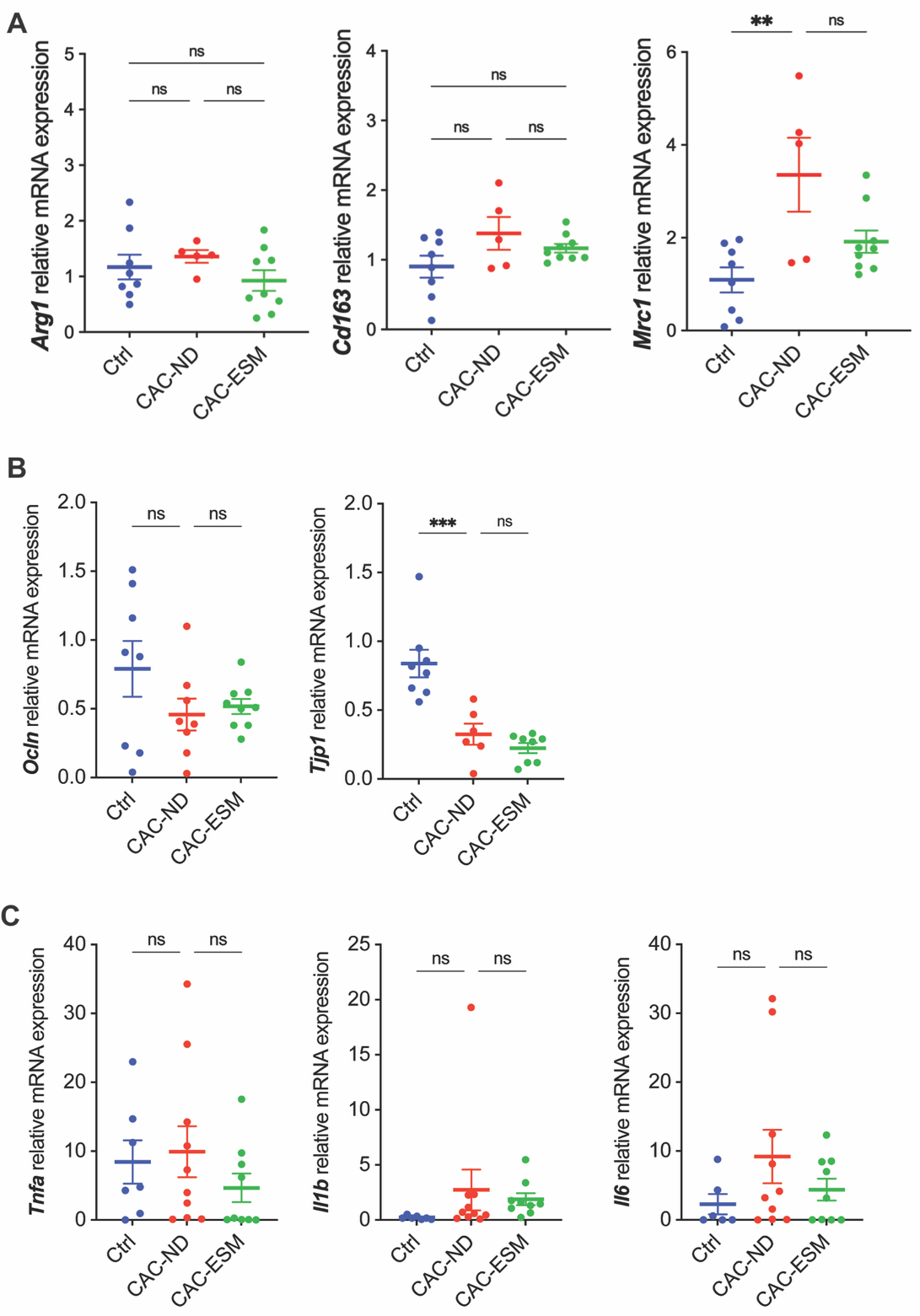
Supplementary Figure S1: Effects of ESM supplementation on** **the expression of colonic mucosal M2-like macrophage markers, epithelial tight junction genes, and hepatic inflammatory cytokines.** (**A**) Relative mRNA expression of genes associated with M2-like macrophage (*Arg1*, *Cd163*, *Mrc1*) in the colonic mucosa. (**B**) Relative *Ocln* and *Tjp1* mRNA expression in the colonic mucosa. (**C**) Relative hepatic *Tnfa, Il1b* and *Il6* mRNA expression. All data are from control (Ctrl), CAC-ND and CAC-ESM mice. Statistical analysis was performed using one-way ANOVA followed by Tukey’s test. Data are expressed as mean ± SEM. ***P* < 0.01, ns, not significant. Symbols represent individual mice. *n* = 5–9 per group.

**Supplementary Figure S2: ESM supplementation modulates cecal microbiota composition at the phylum and family levels.** (**A** and **B**) Relative abundance of gut bacteria in cecal contents of Ctrl (*n* = 9), CAC-ND (*n* = 7), and CAC-ESM (*n* = 7) mice at the phylum (**A**) and family levels (**B**).

**Supplementary Figure S3: ESM supplementation rescues microbiome disruption in CAC mice.** (**A–C**) Relative abundance of the top 10 most abundant gut bacterial genera in Ctrl (*n* = 9) (**A**), CAC-ND (*n* = 7) (**B**), and CAC-ESM (*n* = 7) (**C**) groups. Only the top 10 bacterial taxa within each group were selected for visualization. (**D**) Pathway analysis was performed using PICRUSt2, and significantly altered pathways identified by ANCOM were shown. Dots represent outlier samples.

**Supplementary Table 1: Diet composition**

| **Component** | **Control Diet (% w/w)** | **8% ESM Supplemented Diet (% w/w)** |
| --- | --- | --- |
| Casein | 20.0 | 16.3 |
| L-cystine | 0.3 | 0.3 |
| β-corn starch | 39.7 | 35.4 |
| α-corn starch | 13.2 | 13.2 |
| Sucrose | 10.3 | 10.3 |
| Soybean oil | 7.0 | 7.0 |
| Cellulose | 5.0 | 5.0 |
| Mineral mixture  (AIN93G-Mix) | 3.5 | 3.5 |
| Vitamin mixture  (AIN93G-Mix) | 1.0 | 1.0 |
| ESM powder | 0.0 | 8.0 |
| Total | 100 | 100 |

AIN-93G prescription (Oriental Yeast Co., Ltd., Tokyo, Japan).

ESM: Eggshell membrane.

**Supplementary Table 2: Primers for real-time PCR analysis.**

| **mRNA (mouse)** |  |  | **Primer sequence** |  |
| --- | --- | --- | --- | --- |
| *Rplp1* | F | 5′ | ATCTACTCCGCCCTCATCCT | 3′ |
|  | R | 5′ | CAGATGAGGCTCCCAATGTT | 3′ |
| *Actb* | F | 5′ | ATGACCCAGATCATGTTTGA | 3′ |
|  | R | 5′ | TACGACCAGAGGCATACAG | 3′ |
| *Il1b* | F | 5′ | TGGTGTGTGACGTTCCCATTAG | 3′ |
|  | R | 5′ | GGTTGATATTCTGTCCATTGAGGTG | 3′ |
| *Il6* | F | 5′ | CTGGCTTTGTCTTTCTTGTT | 3′ |
|  | R | 5′ | ATTTCCTCTGGTCTTCTGG | 3′ |
| *Tnfa* | F | 5′ | GGCAGGTCTACTTTGGAGTCATTGC | 3′ |
|  | R | 5′ | ACATTCGAGGCTCCAGTGAATTCGG | 3′ |
| *Itgam* | F | 5′ | CTTCGGGCAGTCTCTGAGTG | 3′ |
|  | R | 5′ | CCTCCCCAGCATCCTTGTTT | 3′ |
| *S100a8* | F | 5′ | TCCTTGCGATGGTGATAAAAGTG | 3′ |
|  | R | 5′ | CCCAGCCCTAGGCCAGAA | 3′ |
| *S100a9* | F | 5′ | CACAAACCAGGACAATCAGC | 3′ |
|  | R | 5′ | GCCATTCCCTTTAGACTTGG | 3′ |
| *Saa3* | F | 5′ | TGCCATCATTCTTTGCATCTTGA | 3′ |
|  | R | 5′ | CCGTGAACTTCTGAACAGCCT | 3′ |
| *Nos2* | F | 5′ | TTACGTCCATCGTGGACAGC | 3′ |
|  | R | 5′ | TGGGCTGGGTGTTAGTCTTA | 3′ |
| *Arg1* | F | 5′ | CTGGTTGTCAGGGGAGTGTT | 3′ |
|  | R | 5′ | GTGAAGAACCCACGGTCTGT | 3′ |
| *Cd163* | F | 5′ | GGTGGACACAGAATGGTTCTTC | 3′ |
|  | R | 5′ | CCAGGAGCGTTAGTGACAGC | 3′ |
| *Mrc1* | F | 5′ | GAGCCCACAACAACTCCTGA | 3′ |
|  | R | 5′ | TCGCCAGCTCTCCACCTATA | 3′ |
| *Cldn-1* | F | 5′ | CCTGCCCCAGTGGAAGATTTACT | 3′ |
|  | R | 5′ | GTGCTTTGCGAAACGCAGGACAT | 3′ |
| *Ocln* | F | 5′ | GCGATCATACCCAGAGTCTTTC | 3′ |
|  | R | 5′ | GGTGTCTCTAGGTTACCATTGC | 3′ |
| *Tjp1* | F | 5′ | CCATGACTCCTGACGGTTGGTCTT | 3′ |
|  | R | 5′ | CGGATCTCCAGGAAGACACTTGT | 3′ |
